# Incidental Diffuse Splenic Polyangiomatosis With Long-Term Radiologic Stability: A Case Report

**DOI:** 10.7759/cureus.108606

**Published:** 2026-05-10

**Authors:** Paola Vanessa Sosa Sarmiento, Kevin A Montoya, Roberto P Guerrero, Yelka Matos Furones, Jose I Gamboa Arisso

**Affiliations:** 1 Geriatrics, University of Valle, Cali, COL; 2 Health Sciences, National Autonomous University of Honduras, San Pedro Sula, HND; 3 Internal Medicine, Loyola MacNeal Hospital, Berwyn, USA; 4 Neurology, Alcanza Clinical Research, Woodstock, USA; 5 General Medicine, Jimmy Hirtzel Hospital, Miami, USA

**Keywords:** benign vascular proliferation, diffuse splenic vascular lesions, incidental splenic findings, mri characterization, multinodular splenomegaly, splenic polyangiomatosis

## Abstract

Splenic polyangiomatosis is an exceptionally rare benign vascular proliferation characterized by multiple vascular channels that may replace large portions of the splenic parenchyma. Most reported cases are discovered incidentally, as the condition is often asymptomatic and identified during imaging performed for unrelated reasons. We report the case of a 46-year-old asymptomatic woman in whom splenomegaly was detected during a routine clinical evaluation. Cross-sectional imaging with CT and MRI revealed marked splenomegaly with innumerable solid and cystic nodular lesions replacing more than 80% of the splenic parenchyma, with stable radiologic features over several years. A comprehensive diagnostic approach integrating clinical findings, laboratory evaluation, and multimodality imaging supported the diagnosis of benign splenic polyangiomatosis. This case underscores the importance of clinicoradiologic correlation in distinguishing this rare benign entity from other vascular, infectious, inflammatory, infiltrative, and malignant causes of multinodular splenic disease, and highlights the relevance of long-term imaging stability in guiding conservative management.

## Introduction

Splenic polyangiomatosis, also known as diffuse splenic hemangiomatosis, is an exceptionally rare benign vascular disorder characterized by the proliferation of multiple vascular channels that partially or completely replace the normal splenic parenchyma [1]. It is part of the broader spectrum of benign splenic vascular lesions, which are being recognized with increasing frequency due to advances and wider use of cross-sectional imaging [2-4].

The pathogenesis of splenic polyangiomatosis remains poorly understood. Current evidence suggests a congenital vascular malformation or a benign proliferative vascular process involving the spleen [1,2]. Owing to its rarity, published data are limited primarily to isolated case reports and small case series rather than comprehensive epidemiologic studies [1].

The true incidence and prevalence of this condition are unknown, with no population-based data available for either the general population or specific demographic groups, including Hispanic patients [1,2]. Most cases are discovered incidentally during imaging performed for unrelated clinical indications, as patients are often asymptomatic [1-4].

Multimodality imaging, particularly CT and MRI, plays a pivotal role in the noninvasive characterization of splenic vascular lesions and in refining the differential diagnosis [2-5]. This report describes an incidental case of diffuse splenic polyangiomatosis with long-term radiologic stability, highlighting the importance of clinicoradiologic correlation and contributing to the limited literature on the imaging features and natural history of this rare benign splenic entity.

## Case presentation

A 46-year-old woman with no significant past medical history presented to the internal medicine clinic for a routine check-up. She was completely asymptomatic, denying cardiopulmonary, gastrointestinal, genitourinary, constitutional, or dermatologic symptoms. Vital signs were within normal limits (blood pressure 120/84 mmHg, heart rate 60 bpm, respiratory rate 16 breaths per minute, temperature 97.3°F). Abdominal examination revealed splenomegaly, which was non-tender. Examination of the skin and mucous membranes showed no vascular lesions, such as angiomas or telangiectasias, and no hemorrhagic manifestations, including petechiae, purpura, or spontaneous ecchymoses. No peripheral lymphadenopathy was appreciated on cervical, axillary, or inguinal examination, and no stigmata of chronic liver disease were present.

Upon further questioning, the patient recalled being told of a similar finding in the past, though she did not remember specific details. She subsequently provided prior imaging studies from 2021 and 2023 performed at an outside institution.

**Figure 1:**
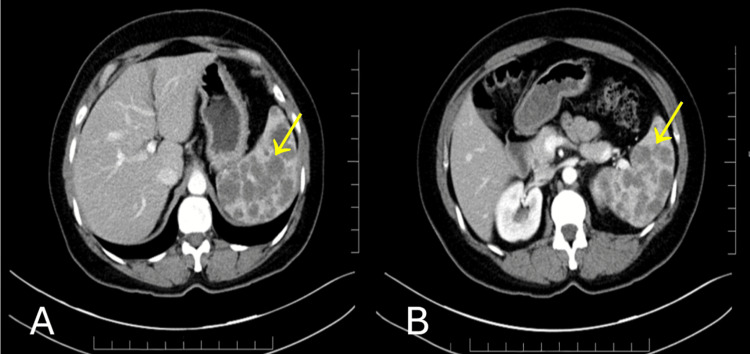
Contrast-enhanced CT images (September 27, 2021). A. Axial abdominal CT image demonstrating diffuse splenomegaly with multiple irregular low‑attenuation splenic lesions (yellow arrow), consistent with diffuse parenchymal involvement. B. Axial contrast-enhanced CT image at a slightly inferior level, confirming innumerable low‑attenuation splenic lesions (yellow arrow) and further illustrating the extent of splenic involvement.

A contrast-enhanced CT of the abdomen dated September 27, 2021, demonstrated diffuse splenomegaly measuring approximately 15.9 cm, with innumerable irregular low-density lesions throughout the splenic parenchyma. The liver showed diffuse decreased attenuation consistent with hepatic steatosis, while the pancreas, adrenal glands, kidneys, biliary tree, bowel, and major abdominal vessels were unremarkable. No lymphadenopathy, free fluid, or other acute intra-abdominal abnormalities were identified. The radiologist noted that a prior abdominal ultrasound from January 19, 2021, had described several simple splenic cysts, although not to the extent seen on CT; however, the ultrasound images were not available for review. Given the multiplicity and incomplete characterization of the lesions on this single-phase CT, contrast-enhanced abdominal MRI was recommended (Figure 1).

**Figure 2:**
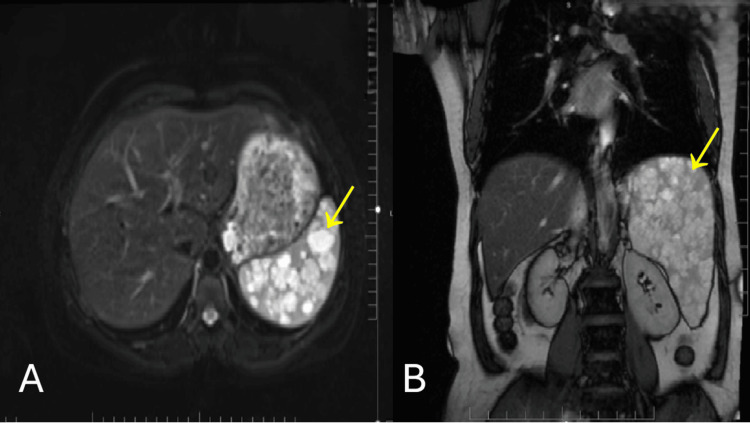
Contrast-enhanced abdominal MRI (December 8, 2021). A. Axial post‑contrast MRI demonstrating marked splenomegaly with innumerable solid and cystic lesions nearly replacing the splenic parenchyma, with heterogeneous progressive enhancement (yellow arrow). B. Coronal non‑contrast MRI from the same examination showing diffuse nodular replacement of the spleen, while the remaining abdominal organs appear preserved (yellow arrow).

A contrast-enhanced abdominal MRI performed on December 8, 2021, confirmed marked splenomegaly measuring approximately 15.6 cm, with innumerable solid and cystic lesions nearly replacing the entire splenic parenchyma. Most lesions demonstrated T1 isointensity to slight hypointensity, predominantly T2 hyperintense signal, and progressive heterogeneous post-contrast enhancement, with the largest measuring 2-3 cm. No lymphadenopathy, vascular abnormalities, or additional intra-abdominal pathology was identified. The liver showed probable mild steatosis, and the remaining abdominal organs were unremarkable. Compared with the CT from September 27, 2021, the findings were grossly stable. The radiologic differential diagnosis included multifocal littoral cell angiomas, atypical hemangiomas, lymphangiomas, or hamartomas, and short-term follow-up imaging was recommended given the absence of symptoms or risk factors for splenic malignancy (Figure 2).

**Figure 3:**
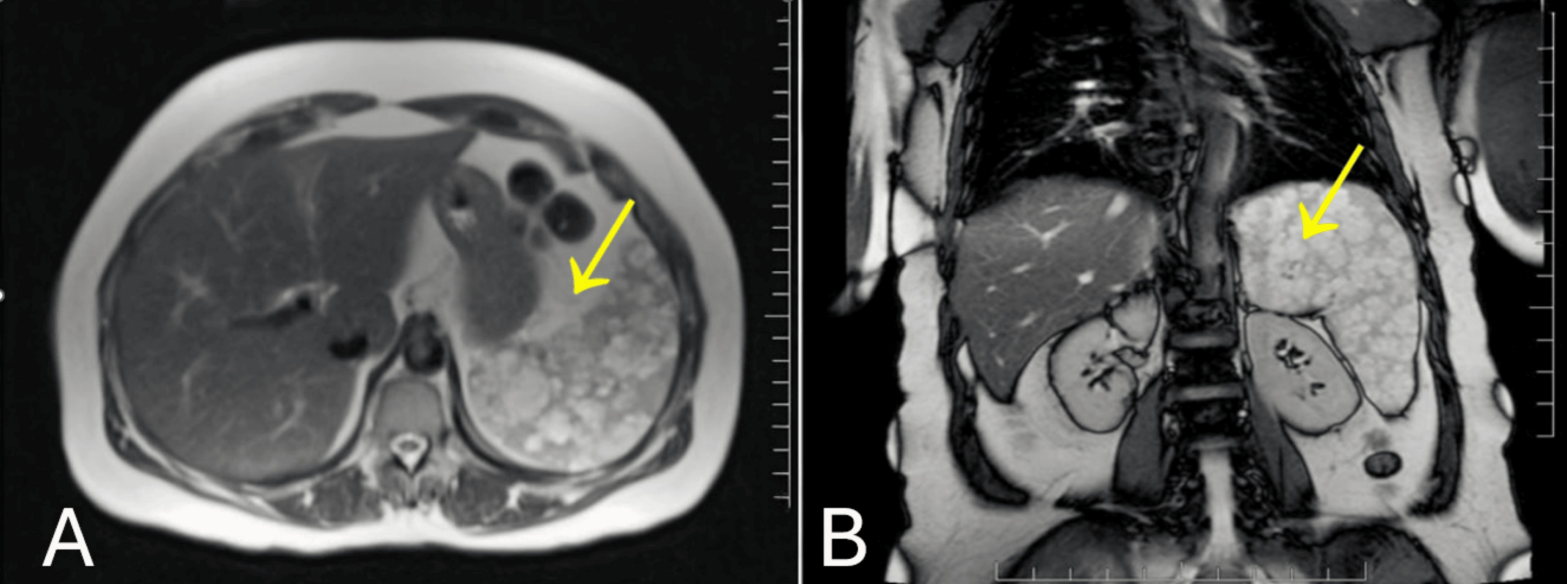
Abdominal MRI (February 2, 2023). A. Axial MRI demonstrating diffuse multinodular replacement of the spleen with similar signal characteristics to prior studies, including scattered simple cystic lesions, and overall imaging stability compared with examinations dating back to 2021 (yellow arrow). B. Coronal MRI from the same study showing moderate to marked splenomegaly with more than 100 partially confluent nodular lesions replacing approximately 80% of the splenic parenchyma (yellow arrow).

A follow-up abdominal MRI performed on February 2, 2023, demonstrated moderate to marked splenomegaly measuring approximately 17 cm, with more than 100 partially confluent nodular lesions replacing at least 80% of the splenic parenchyma. The lesions measured approximately 2-3 cm, appeared T1 hypointense, showed variable but predominantly T2 hyperintense signal, and exhibited progressive post-contrast enhancement without restricted diffusion. A subset of lesions demonstrated simple cystic characteristics, measuring up to 2.3 cm. Mild hepatic steatosis persisted. Overall, the imaging appearance remained stable compared with prior studies dating back to 2021 (Figure 3).

Laboratory evaluation revealed a complete blood count with normal leukocyte white blood cell (WBC) and platelet counts, with mild macrocytosis noted, reflected by an elevated mean corpuscular volume (MCV). Lactate dehydrogenase (LDH), C‑reactive protein (CRP), erythrocyte sedimentation rate (ESR), and hemoglobin A1c (HbA1c) were all within normal limits (Table 1). Infectious etiologies were excluded, including hepatitis A, B, and C; human immunodeficiency virus (HIV) (Table 1); chlamydia; gonorrhea; and Helicobacter pylori.

**Table 1:** Laboratory results.

Parameter	Reference Range	Result	Interpretation
White Blood Cell	4.0–11.0 ×10³/µL	4.1	Within normal limits
Hemoglobin	12.0–16.0 g/dL	13.4	Normal
Platelets	150–400 ×10³/µL	193	Normal
Mean Corpuscular Volume	82.0–97.0 fL	97.2	Mild macrocytosis
Lactate dehydrogenase	100–220 U/L	107	Normal
C‑reactive protein	<5 mg/L	<5	Normal
Erythrocyte Sedimentation Rate	0–20 mm/hr	10	Normal
Hemoglobin A1c	<5.7 %	5.5	Normal
Hepatitis A, B, C	Negative	Negative	No infection
Human Immunodeficiency Virus	Non-reactive	Non-reactive	No infection

Additional history included ongoing gynecologic follow-up for a positive high-risk human papillomavirus (HPV) test (negative for subtypes 16, 18, and 45). A screening mammogram performed in December 2025 was classified as breast imaging reporting and data system (BIRADS) 1 [6]; however, the original imaging was not available for independent review and therefore could not be included in the case presentation.

Given the patient’s long‑term radiologic stability, absence of symptoms, and normal laboratory profile, the overall picture was most consistent with benign splenic polyangiomatosis. She was referred to general surgery for discussion of management options, including elective splenectomy versus conservative monitoring. Moderate physical activity was considered safe and recommended as part of general health optimization, while avoiding high‑impact or contact activities due to the presence of splenomegaly, and vitamin D supplementation was initiated.

## Discussion

The clinicoradiologic features in this case are most consistent with benign splenic polyangiomatosis, a rare multifocal benign vascular proliferation, rather than alternative vascular, infectious, inflammatory, infiltrative, or malignant causes of splenomegaly. Splenomegaly carries a broad differential diagnosis that includes hematologic disorders, congestive states, infectious diseases, inflammatory and autoimmune conditions, infiltrative processes, and benign or malignant splenic masses [7]. In this patient, the combination of marked splenomegaly, innumerable T2‑hyperintense nodules with progressive post‑contrast enhancement, long‑term radiologic stability, and the absence of systemic symptoms or laboratory abnormalities substantially narrows the diagnostic possibilities.

Among benign vascular tumors, littoral cell angioma was considered but deemed unlikely because it is frequently associated with anemia, thrombocytopenia, or autoimmune disease, none of which were present in this case [7]. In addition, littoral cell angioma typically preserves areas of normal splenic parenchyma, in contrast to the near‑complete parenchymal replacement observed here. Multiple hemangiomas were also considered, but are usually few in number and demonstrate peripheral nodular enhancement rather than the diffuse, homogeneous vascular pattern seen in this patient [7-10]. Hamartomas are most often solitary lesions, while lymphangiomas typically appear as multiloculated cystic masses with minimal enhancement, both inconsistent with the imaging findings [4]. Sclerosing angiomatoid nodular transformation (SANT) was excluded because it usually presents as a solitary lesion with a characteristic spoke‑wheel enhancement pattern, rather than innumerable uniform nodules [4].

Non‑vascular benign causes of splenomegaly were similarly excluded. Splenic abscesses classically present with rim‑enhancing lesions accompanied by fever, leukocytosis, or an immunocompromised state, none of which were present [10-12]. Granulomatous infections such as tuberculosis or histoplasmosis typically produce calcified or poorly defined nodules and are often associated with pulmonary or systemic manifestations, which were not observed in this patient [13]. Inflammatory and autoimmune conditions, including sarcoidosis, were considered unlikely given the absence of lymphadenopathy, systemic symptoms, or laboratory abnormalities; sarcoidosis more commonly presents with small hypodense splenic nodules and mediastinal lymphadenopathy [11-13]. Congestive etiologies such as portal hypertension or splenic vein thrombosis were excluded based on normal hepatic function, absence of collateral vessels, and unremarkable vascular imaging [7]. Structural lesions, including cysts, pseudocysts, or echinococcal disease, were not favored given the predominantly solid and vascular nature of the lesions [10].

Malignant causes such as lymphoma or metastatic disease were effectively excluded by the absence of B symptoms, normal blood counts, lack of lymphadenopathy, and prolonged radiologic stability-features incompatible with untreated malignancy [9-13].

Although the patient had a history of high‑risk HPV infection, there is no evidence linking HPV to benign vascular splenic proliferations, including polyhemangiomatosis. HPV demonstrates a strong tropism for squamous epithelium rather than endothelial tissue, and no published studies have identified HPV deoxyribonucleic acid (DNA) or related oncogenic pathways in splenic vascular lesions. Accordingly, the patient’s HPV status is considered incidental and unrelated to the splenic findings [14].

Taken together, the diffuse multinodular vascular pattern, progressive enhancement, T2 hyperintensity, long‑term radiologic stability, and absence of systemic or laboratory abnormalities strongly support benign splenic polyangiomatosis as the most plausible diagnosis. This entity represents a benign multifocal vascular proliferation that may extensively replace splenic parenchyma while remaining clinically silent, as demonstrated in this case.

Histologic confirmation was not pursued because the patient remained asymptomatic and hemodynamically stable and did not meet criteria for urgent splenectomy. Consistent with current management strategies for incidentally discovered benign‑appearing splenic lesions, the patient was referred for elective surgical evaluation to assess the need for splenectomy in a non‑emergent setting should symptoms or disease progression occur [15].

Management of incidentally detected benign splenic vascular lesions relies on individualized risk assessment and longitudinal surveillance. In patients with marked splenomegaly, counseling regarding avoidance of high‑impact activities is prudent due to the theoretical risk of traumatic rupture. However, spontaneous rupture in benign vascular proliferations is exceedingly rare. Elective splenectomy is generally reserved for patients who develop symptoms, show interval growth or atypical imaging features, or require histologic confirmation. In asymptomatic patients with stable imaging findings, as in this case, periodic follow‑up remains a safe and evidence‑supported approach.

Limitations

A key limitation of this report is its single‑patient nature, which inherently restricts the generalizability of the imaging-clinical correlations described. Although the radiologic features strongly supported a benign multifocal vascular process, the absence of histopathologic confirmation remains another important limitation. The patient did not meet criteria for urgent splenectomy and remained hemodynamically stable and asymptomatic throughout evaluation; therefore, tissue diagnosis was not pursued. Instead, she was appropriately referred to general surgery for outpatient assessment to determine whether an elective splenectomy would be indicated based on future clinical evolution or symptom development. This lack of histologic confirmation limits the ability to classify the lesion definitively but reflects real‑world clinical decision‑making in cases where imaging findings are benign‑appearing and the risks of surgery outweigh the immediate diagnostic benefit.

## Conclusions

This case emphasizes the importance of detailed clinicoradiologic correlation in the evaluation of diffuse multinodular splenic lesions, particularly for rare benign entities such as splenic polyangiomatosis. The presence of marked splenomegaly, innumerable T2‑hyperintense nodules with progressive enhancement, sustained radiologic stability across multiple imaging modalities, absence of systemic symptoms, and normal laboratory findings supported a benign vascular process and enabled systematic exclusion of infectious, inflammatory, infiltrative, and malignant conditions. Although histopathologic confirmation was not obtained, the convergence of characteristic imaging features with long‑term clinical stability provided sufficient diagnostic confidence. This case illustrates the value of a structured differential diagnostic approach and supports conservative management with surveillance in asymptomatic patients with stable, benign‑appearing splenic lesions, thereby avoiding unnecessary invasive intervention.
